# Supplementary material for: Investigating the application of IoT mobile app and healthcare services for diabetic elderly: A systematic review
Source: PLoS One. 2025 Apr 15;20(4):e0321090. doi: 10.1371/journal.pone.0321090 (PMC11999127; doi:10.1371/journal.pone.0321090)
Supplement: S1 File — (ZIP) [file pone.0321090.s001.zip › S1 File/1-PRISMA checklist-18- sep-2024.docx]

| **Section and Topic** | **Item #** | **Checklist item** | **Location where item is reported** |
| --- | --- | --- | --- |
| **TITLE** | | |  |
| Title | 1 | Investigating the Application of IoT Mobile App and Healthcare Services for Diabetic Elderly: A Systematic Review |  |
| **ABSTRACT** | | |  |
| Abstract | 2 | As the prevalence of diabetes increases among the elderly population, effective management becomes increasingly crucial. IoT mobile applications offer promising solutions for diabetes care by providing real-time monitoring, medication management, and lifestyle support. This systematic literature review investigated the potential applications and challenges of IoT mobile applications in managing diabetes among elderly patients. Three databases including Scopus, Web of Science, and IEEE were systematically searched; 29 articles were screened in the final analysis process. Key results presented that the application of mobile apps include blood glucose monitoring, medication adherence, and promotion of physical activity and dietary control. Mobile app devices such as continuous glucose monitors and smart pill dispensers significantly improve glycemic control and medication adherence rates. However, several challenges impede their effective implementation. Technical issues such as interoperability, data security, and device reliability are major concerns. User adoption is hindered by the cognitive and physical limitations of elderly patients, necessitating simplified interfaces and adequate training. Despite these challenges, IoT mobile applications hold significant potential for improving diabetes management in elderly patients, leading to better health outcomes and quality of life. This review highlights the need for collaborative efforts to overcome barriers and fully realize the benefits of IoT technologies in diabetic elderly healthcare services. |  |
| **INTRODUCTION** | | |  |
| Rationale | 3 | IoT applications for diabetic elderly management still face several issues nowadays. Technical challenges such as interoperability between devices, data security and privacy concerns, and the need for reliable wireless connectivity are significant barriers, necessitating user-friendly designs and comprehensive training and support through this review. |  |
| Objectives | 4 | The purpose of this systematic review paper is to investigate the current and development status of IoT mobile applications and healthcare services designed for diabetic elderly patients and to explore the benefits, challenges, and potential future directions of these technologies, emphasizing their impact on patient outcomes and quality of life. |  |
| **METHODS** | | |  |
| Eligibility criteria | 5 | Inclusion criteria:  (a) Articles include healthcare mobile applications as technology for diabetic elderly;  (b) Articles focus on elderly’s healthcare;  (c) Access to the full articles.  Exclusion criteria:   1. IoT mobile application but not healthcare application for elderly; 2. Mobile application does not focus on healthcare; 3. Healthcare mobile application design not for diabetic elderly; 4. Inaccessibility to full-text articles; |  |
| Information sources | 6 | Scopus, WoS, and IEEE |  |
| Search strategy | 7 | Present the full search strategies for all databases, registers and websites, including any filters and limits used.  Scopus database (<https://www-scopus-com.eresourcesptsl.ukm.remotexs.co/search/form.uri?display=advanced>)  Advanced search query string: TITLE-ABS-KEY ("diabete* old*" OR "diabete* elder*" OR "diabete* aged" OR "diabete* senior*") AND TITLE-ABS-KEY ("mobile app*" OR IoT OR application* OR mobile OR app* OR “smart technolog*” OR “digital solution*”) AND TITLE-ABS-KEY (healthcare OR "health care" OR service* OR “chronic disease management*”)  Filters: (Years range from 2014- 2024)  WoS database (https://www-webofscience-com.eresourcesptsl.ukm.remotexs.co/wos/woscc/advanced-search)  Advanced search query string: (TS=("diabete* old*" OR "diabete* elder*" OR "diabete* aged" OR "diabete* senior*") AND TS=("mobile app*" OR IoT OR application* OR mobile OR app* OR “smart technolog*” OR “digital solution*”)AND TS=(healthcare OR "health care" OR service*))  Filters: (Years range from 2014- 2024)  IEEE database (<https://ieeexplore-ieee-org.eresourcesptsl.ukm.remotexs.co/search/advanced>)  Advanced search query string: ("diabete* old*" OR "diabete* elder*" OR "diabete* aged" OR "diabete* senior*") AND ("mobile app*” OR IoT OR application* OR mobile OR app*) AND (healthcare OR "health care" OR service* OR “chronic disease management*”)  Filters: (Years range from 2014- 2024) |  |
| Selection process | 8 | To determine if a study met the inclusion criteria, two independent reviewers screened the titles and abstracts of 147 articles after removing duplicates. Articles passing this initial screening were then assessed in full text to confirm eligibility. Discrepancies were resolved through discussion, with a third reviewer consulted if necessary. Quality assessment was conducted using the CASP checklist. Automation tools were used only for managing references and removing duplicates. |  |
| Data collection process | 9 | Two independent reviewers collected data from each report using a standardized extraction form. They worked separately to ensure accuracy, resolving discrepancies through discussion or with a third reviewer if needed. For missing or unclear information, reviewers contacted study investigators. No automation tools were used in the data collection process. |  |
| Data items | 10a | \| SCOPUS \| TITLE-ABS-KEY ("diabete* old*" OR "diabete* elder*" OR "diabete* aged" OR "diabete* senior*") AND TITLE-ABS-KEY ("mobile app*" OR IoT OR application* OR mobile OR app*) AND TITLE-ABS-KEY (healthcare OR "health care" OR service*) \| 79 results \| \| --- \| --- \| --- \| \| WoS \| (TS=("diabete* old*" OR "diabete* elder*" OR "diabete* aged" OR "diabete* senior*") AND TS=("mobile app*" OR IoT OR application* OR mobile OR app*)AND TS=(healthcare OR "health care" OR service*)) \| 61 results \| \| IEEE \| ("diabete* old*" OR "diabete* elder*" OR "diabete* aged" OR "diabete* senior*") AND ("mobile app*” OR IoT OR application* OR mobile OR app*) AND (healthcare OR "health care" OR service*) \| 7 results \| |  |
| Study risk of bias assessment | 11 | Two independent reviewers evaluated the identified articles based on the inclusion and exclusion criteria which are listed in item 5 before. The reviewers removed the articles that did not match the selection criteria. The whole articles were re-examined by authors after twice of title and abstract selection, if the articles that did not match requirements of selection criteria will be removed. Disagreements over decisions were resolved during the period of selection step by authors themselves until an agreement was reached. |  |
| Effect measures | 12 | Individual study quality was assessed using the Critical Appraisal Skills Programme (CASP)’s Quality Appraisal Tool systematic review checklist for all included full-text papers, and the result is presented in the form of a table. CASP is a tool that assists researchers in critically evaluating the quality of research studies. It provides a structured framework for assessing various aspects of study design, methodology, analysis, and reporting, helping users determine the trustworthiness, relevance, and validity of research findings. |  |
| Synthesis methods | 13a | Describe the processes used to decide which studies were eligible for each synthesis (e.g. tabulating the study intervention characteristics and comparing against the planned groups for each synthesis (item #5)).  The quality of those total 29 articles was systematically assessed using the CASP checklist, as presented in the table. The CASP tool provides a structured framework for evaluating research, focusing on key aspects such as study aims, recruitment methods, exposure and outcome measurement, and the handling of confounding factors. In this table, articles are rated using a color-coded system: green indicates the criterion was met, orange suggests uncertainty or incomplete information, and red signifies the criterion was not fulfilled. Most studies demonstrated strong methodological rigor, with green marking across most evaluation criteria. However, some studies were found lacking in addressing confounding factors, as seen by red or orange marks in the "Identified confounding?" and "Addressed confounding?" columns. This evaluation highlights the overall quality of the research while identifying specific areas of methodological concern, providing a clear basis for assessing the reliability and applicability of the findings in the context of a systematic review. Despite some variation in addressing confounding factors, all 29 papers ultimately met the necessary qualifications for inclusion, as they demonstrated sufficient methodological rigor and relevance across the key CASP criteria.  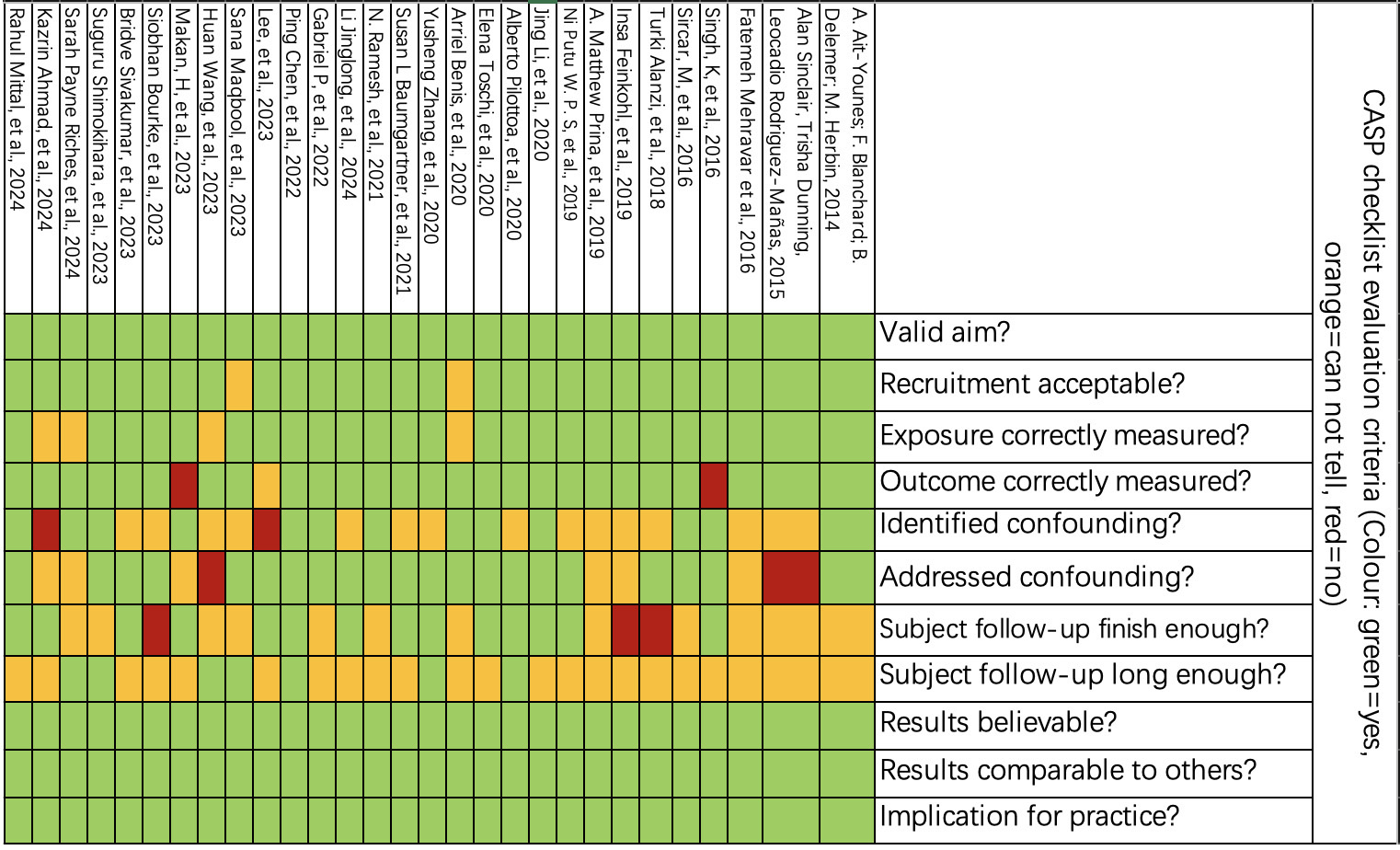 |  |
|  | 13b | Data conversions and coding. |  |
|  | 13c | Results from individual studies and syntheses were tabulated using summary tables that highlighted key outcomes, effect measures, and study characteristics. |  |
|  | 13d | Results were synthesized using a narrative synthesis approach, which was chosen due to the heterogeneity in study designs, populations, and outcomes. This method allowed for a detailed qualitative comparison of findings across studies, highlighting patterns, themes, and relationships within the data without relying on statistical aggregation. |  |
|  | 13e | N/A |  |
|  | 13f | N/A |  |
| Reporting bias assessment | 14 | Although the purpose of this systematic review is descriptive, we assessed the risk of bias and applicability using the Cochrane risk-of-bias tool for randomized trials version 1 (RoB 1). Cochrane risk-of-bias tool is a commonly used tool to assess articles. The tool evaluates six domains, namely: random sequence generation, allocation concealment, blinding of participants and personnel, blind of outcomes assessment, incomplete outcome data and selective reporting. –, ? and + represents either a high, unknown or low risk of bias, respectively. Within each domain, assessments are made for one or more items, which may cover different aspects of the domain, or different outcomes. If any domain was not rated “low”, the overall risk of bias was considered “high”. The assessment is attached to S2 File, (Sheet: Cochrance RoB1) includes the applicability and risk of bias assessment. |  |
| Certainty assessment | 15 | Certainty in the body of evidence for each outcome was assessed using the CASP. |  |
| **RESULTS** | | |  |
| Study selection | 16a | The screening process is illustrated in Figure below (PRISMA flowchart). The systematic search yielded a total of 147 articles, later 33 of those articles were found to be duplicates and were eliminated. As a result, 114 articles still needed to be screened. The first screening process was the title screening, unrelated title articles were removed in this process, accounting for 56 articles; abstracts that did not focus on topics were also removed. Therefore, a final 10 articles were removed. The remaining articles were further screened according to inclusion and exclusion criteria; therefore, another 19 articles were removed. The remaining 29 articles were assessed for eligibility and extracted for literature review.  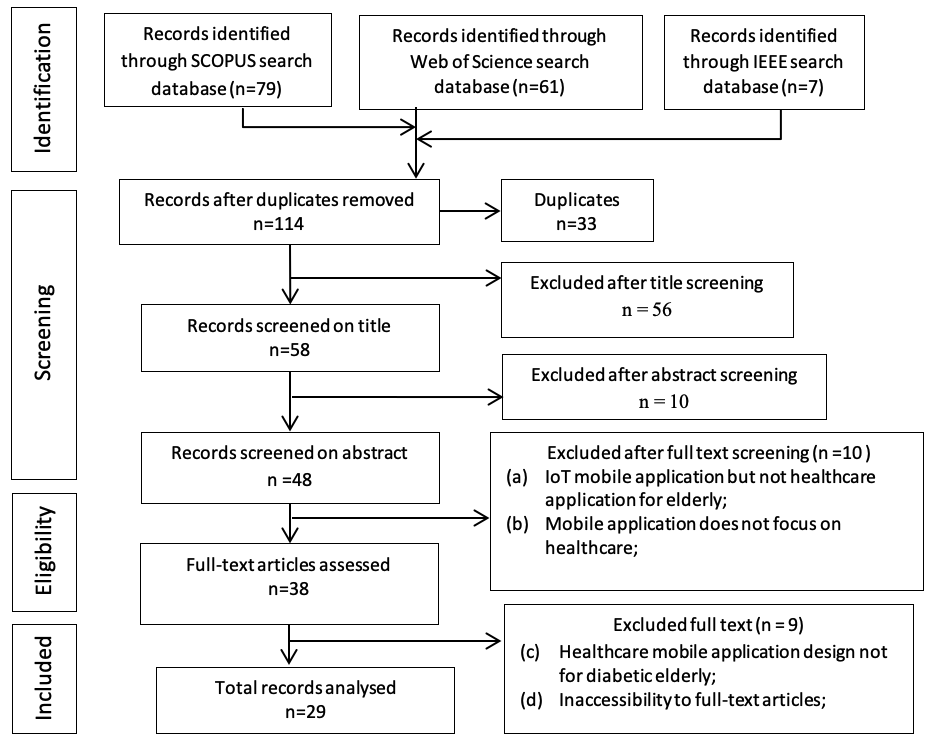 |  |
|  | 16b | The remaining 29 articles were assessed for eligibility and extracted for literature review. Table presents in 13a describes the results of the CASP appraisal for all included articles. |  |
| Study characteristics | 17 | Cite each included study and present its characteristics.  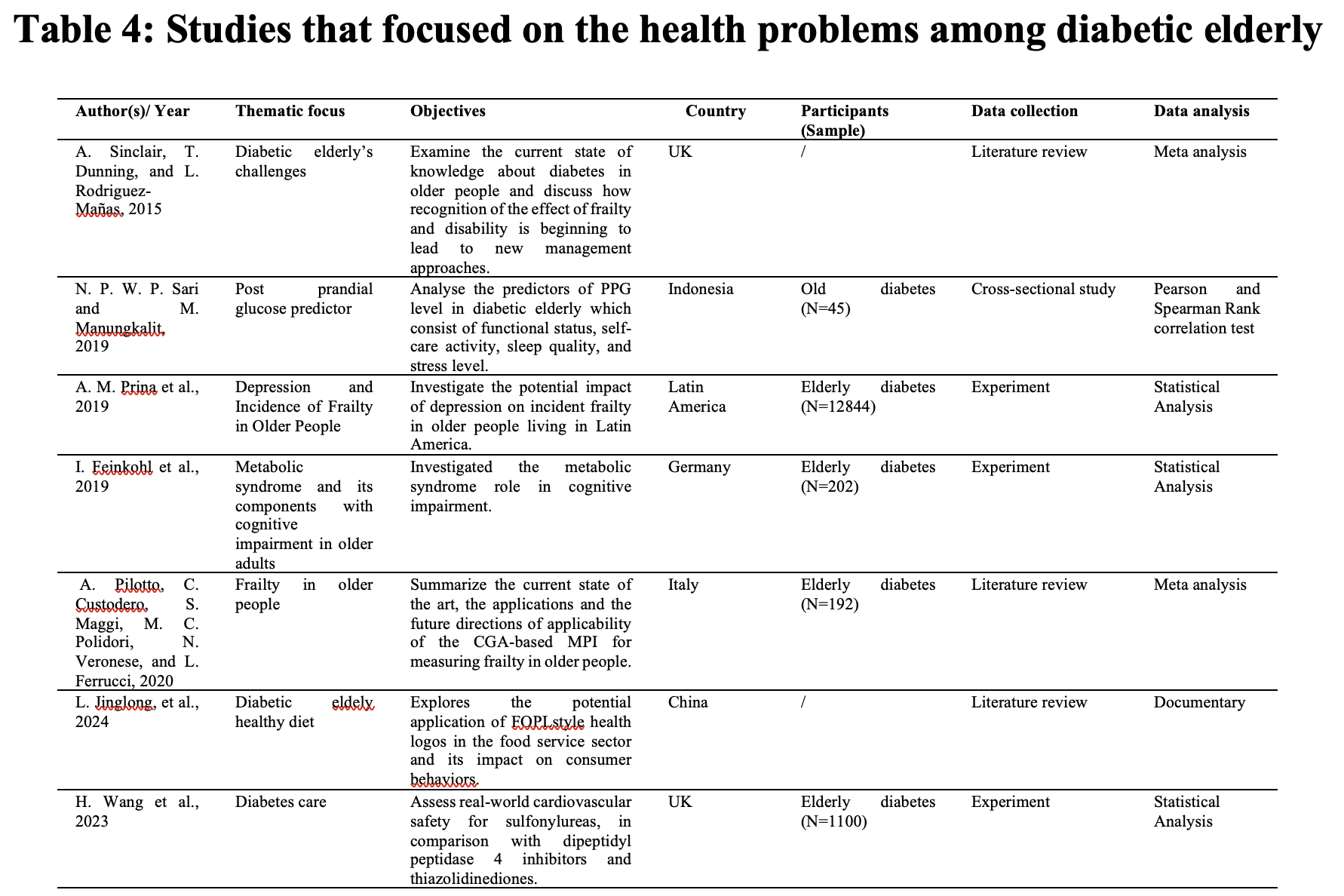  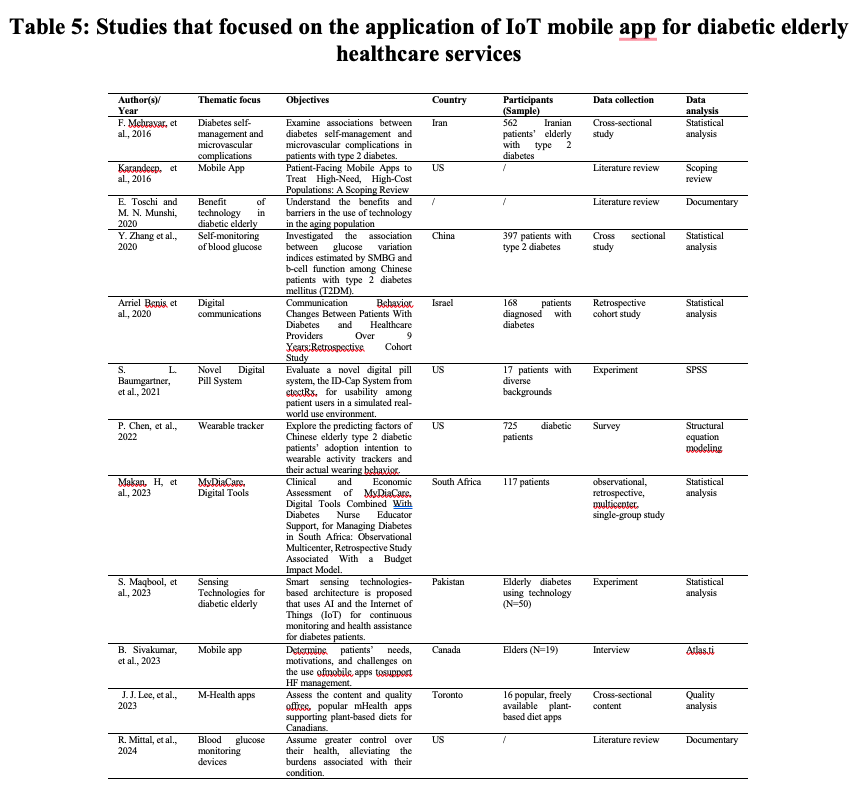  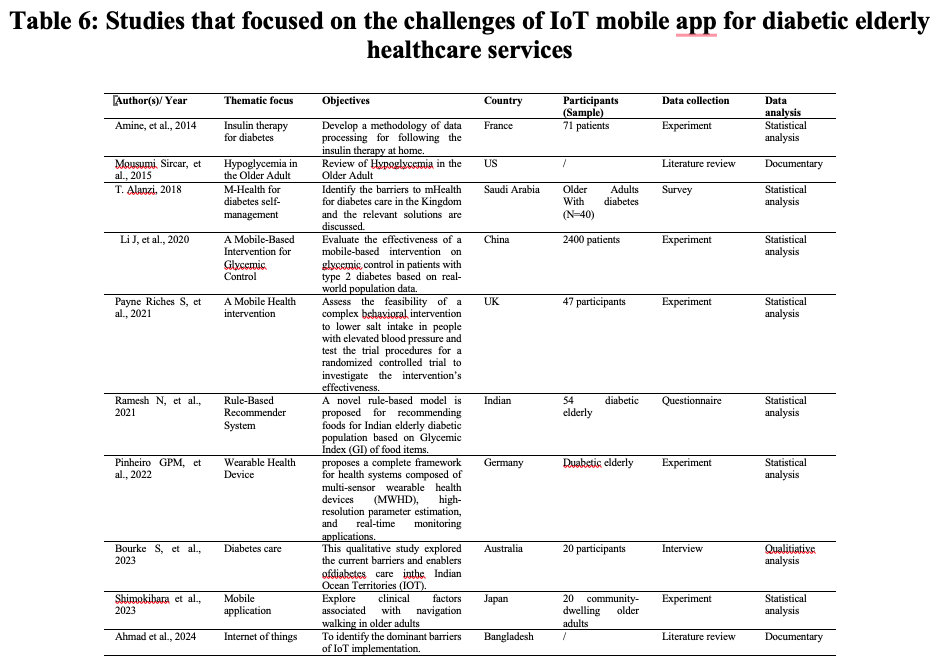 |  |
| Risk of bias in studies | 18 | N/A |  |
| Results of individual studies | 19 | 1. Diabetes poses significant challenges for the elderly, who often neglect their daily care, leading to common issues such as complications, hypoglycemia, frailty, and cognitive impairment. Elderly diabetics face a higher risk of microvascular and macrovascular diseases, with cardiovascular issues and hypoglycemia being particularly prevalent. Long-term diabetes can also lead to frailty and sarcopenia, affecting mobility and functional autonomy. Additionally, diabetes is linked to cognitive decline and conditions like dementia, further complicating daily life and care for elderly patients. 2. IoT mobile applications hold significant potential for improving diabetes management in elderly patients through enhanced monitoring, medication management, and lifestyle support. The benefits of real-time data, personalized care, and increased patient engagement are substantial. However, challenges related to technical issues, user adoption, and data management must be addressed to fully realize the potential of these technologies. 3. IoT mobile applications offer significant potential for enhancing diabetes management in elderly patients through improved monitoring, medication management, and lifestyle support. However, several challenges, including technical issues, user adoption barriers, and data management complexities, must be addressed to fully realize the benefits of these technologies. |  |
| Results of syntheses | 20a | Several attributes were coded and analyzed under this study’s research aims. These characteristics are as follows: The remaining research articles were subjected to quality screening based on the various inclusion and exclusion criteria mentioned in Table 2. Therefore, the final articles were reviewed down to 29 articles as shown in Figure 1. These articles were incorporated into the final step of data coding and analysis. According to the final screened articles, three themes can be classified, they were: (a) Health problems among diabetic elderly; (b) The application of IoT mobile app for diabetic elderly healthcare services; (c) The challenges of IoT mobile app for diabetic elderly healthcare services. |  |
|  | 20b | N/A |  |
|  | 20c | N/A |  |
|  | 20d | N/A |  |
| Reporting biases | 21 | N/A |  |
| Certainty of evidence | 22 | The remaining 29 articles were assessed for eligibility and extracted for literature review. Item 13a table describes the results of the CASP appraisal for all included articles. |  |
| **DISCUSSION** | | |  |
| Discussion | 23a | IoT mobile applications hold significant promise for enhancing diabetes management in elderly patients through improved monitoring, medication management, and lifestyle support. These technologies offer innovative solutions that can facilitate real-time tracking of blood glucose levels, ensure timely medication adherence, and promote healthy lifestyle choices. The potential benefits include better glycemic control, reduced complications, and improved overall quality of life for diabetic elderly individuals. |  |
|  | 23b | The evidence included in the review was limited by literature review, heterogeneity in study designs, potential publication bias, and a lack of focus on elderly-specific challenges. |  |
|  | 23c | The review process was limited by potential selection bias due to reliance on specific databases, possible reviewer bias despite independent assessments, and the absence of automation tools, which could have led to human error or oversight in data extraction and analysis. |  |
|  | 23d | IoT mobile applications hold significant potential for improving diabetes management in elderly patients, leading to better health outcomes and quality of life. This review highlights the need for collaborative efforts to overcome barriers and fully realize the benefits of IoT technologies in diabetic elderly healthcare services. |  |
| **OTHER INFORMATION** | | |  |
| Registration and protocol | 24a | The review was not registered. |  |
|  | 24b | That a protocol was not prepared. |  |
|  | 24c | No amendments to information provided at registration or in the protocol. |  |
| Support | 25 | This research received no external funding. |  |
| Competing interests | 26 | No potential conflict of interest was reported by the author(s). |  |
| Availability of data, code and other materials | 27 | The data that support the findings of this study are available from the corresponding author Li, upon reasonable request. |  |

*From:*  Page MJ, McKenzie JE, Bossuyt PM, Boutron I, Hoffmann TC, Mulrow CD, et al. The PRISMA 2020 statement: an updated guideline for reporting systematic reviews. BMJ 2021;372:n71. doi: 10.1136/bmj.n71
