# Supplementary material for: Investigating the application of IoT mobile app and healthcare services for diabetic elderly: A systematic review
Source: PLoS One. 2025 Apr 15;20(4):e0321090. doi: 10.1371/journal.pone.0321090 (PMC11999127; doi:10.1371/journal.pone.0321090)
Supplement: S1 File — (ZIP) [file pone.0321090.s001.zip › S1 File/2-Detailed inclusion and exclusion criteria.docx]

| **Inclusion Criteria** | **Exclusion Criteria** |
| --- | --- |
| (a) Articles include healthcare mobile applications as technology for diabetic elderly;  (b) Articles focus on diabetic elderly’s healthcare;  (c) Access to the full articles. | 1. IoT mobile application but not healthcare application for diabetic elderly; 2. Mobile application does not focus on healthcare; 3. Healthcare mobile application design not for diabetic elderly; 4. Inaccessibility to full-text articles. |

**Table: Inclusion and exclusion criteria**
