# Supplementary material for: Investigating the application of IoT mobile app and healthcare services for diabetic elderly: A systematic review
Source: PLoS One. 2025 Apr 15;20(4):e0321090. doi: 10.1371/journal.pone.0321090 (PMC11999127; doi:10.1371/journal.pone.0321090)
Supplement: S1 File — (ZIP) [file pone.0321090.s001.zip › S1 File/3-Literature search syntax.docx]

**Table: Search strings from Scopus, WoS and IEEE**

| **SCOPUS** | TITLE-ABS-KEY ("diabete* old*" OR "diabete* elder*" OR "diabete* aged" OR "diabete* senior*") AND TITLE-ABS-KEY ("mobile app*" OR IoT OR application* OR mobile OR app* OR “smart technolog*” OR “digital solution*”) AND TITLE-ABS-KEY (healthcare OR "health care" OR service* OR “chronic disease management*”) |
| --- | --- |
| **WoS** | (TS=("diabete* old*" OR "diabete* elder*" OR "diabete* aged" OR "diabete* senior*") AND TS=("mobile app*" OR IoT OR application* OR mobile OR app* OR “smart technolog*” OR “digital solution*”)AND TS=(healthcare OR "health care" OR service* OR “chronic disease management*”)) |
| **IEEE** | ("diabete* old*" OR "diabete* elder*" OR "diabete* aged" OR "diabete* senior*") AND ("mobile app*” OR IoT OR application* OR mobile OR app* OR “smart technolog*” OR “digital solution*”) AND (healthcare OR "health care" OR service* OR “chronic disease management*”) |
