# Supplementary material for: Investigating the application of IoT mobile app and healthcare services for diabetic elderly: A systematic review
Source: PLoS One. 2025 Apr 15;20(4):e0321090. doi: 10.1371/journal.pone.0321090 (PMC11999127; doi:10.1371/journal.pone.0321090)
Supplement: Attachment — Submitted filename: 4-Response to Reviewers.doc [file pone.0321090.s004.doc]

|  | **[PONE-D-24-34100] - [EMID:adb902876390f92c]** |
| --- | --- |
|  | **RESPONSE TO REVIEWERS** |

**Response to Reviewer’s Comments**

I am grateful for your insightful comments. I have been able to incorporate changes to reflect all of the suggestions provided by you before. I have also highlighted the changes in the revised manuscript with track changes for your review.

| **No** | **Comments** | **Response** | **Page** |
| --- | --- | --- | --- |
| 1. | **Reviewer #1**: The manuscript titled "Investigating the Application of IoT Mobile App and Healthcare Services for Diabetic Elderly: A Systematic Review" effectively addresses a critical health issue but has some notable weaknesses. Firstly, the scope of the study is somewhat limited by the relatively small sample size of 18 articles reviewed. This constraint restricts the generalizability of the findings, as the study only captures a narrow snapshot of the available literature on IoT mobile applications in healthcare for the diabetic elderly.  Moreover, the study primarily focuses on positive outcomes, while not giving sufficient attention to more granular aspects of the challenges, particularly those beyond technical interoperability and data security issues. | 1.Thank you for your valuable comments. I acknowledge that the limited number of articles included in the initial review may have impacted the generalizability of the findings. The primary reason for the smaller sample size was the rigorous inclusion criteria we applied to ensure the relevance and quality of the studies, which led to the exclusion of many articles that did not specifically focus on IoT. However, I agree that a larger sample size could provide a more comprehensive overview. To address this, I have expanded the search criteria by adding additional keywords, which has allowed for a more extensive and updated systematic search and screening process (**Page 6**). As a result, the sample size has now been increased to 29 articles. This broader sample enables us to offer a more in-depth analysis, with greater focus on the applications, challenges, and nuances related to IoT mobile applications in healthcare for the diabetic elderly. Additionally, I have highlighted this limitation in the revised manuscript, noting the sample size of 29 articles in the conclusion (**page 21, line 8**).  2. Thank you for your valuable suggestion to provide a more in-depth discussion of the challenges. In response, I have revised the manuscript to include additional paragraphs that address a broader range of challenges. These now encompass not only technical barriers such as interoperability and data security but also issues related to usability, user engagement, and the involvement of policymakers in promoting successful adoption. This expanded discussion can be found in the revised manuscript on **pages 15 to 18**, with corresponding updates made in the abstract and conclusion sections as well. | 1. Page 6, [Result] part, line 14; Page 21, line 8 begin.  2.Page 15-18, [The challenges og IoT mobile app for diabetic elderly healthcare services] part. |
| 2. | **Reviewer #1**: Secondly, the methodology employed for selecting and reviewing the articles could have been more rigorous. Although the paper mentions the use of CASP's Quality Appraisal Tool, it does not provide detailed insights into how each study was critically evaluated. This omission weakens the reliability of the quality assessment, leaving readers with limited understanding of the varying quality of the studies included.  Additionally, while the discussion highlights key challenges such as data privacy and usability issues for elderly patients, it lacks depth in exploring how these challenges can be systematically addressed through future research and implementation. | 1. Thank you for pointing out the need for a more detailed explanation of how the CASP Quality Appraisal Tool was applied. In response, I have revised the methodology section to provide a clearer, step-by-step description of how each study was critically evaluated using the CASP tool. This addition enhances the transparency and rigor of the quality assessment process. You can find the updated explanation in the revised manuscript on **pages 6-7**.  2. Thank you for highlighting the need for a more detailed exploration of how key challenges, such as data privacy and usability, can be addressed through future research and implementation. In response, I have expanded the discussion section to include potential strategies and frameworks for addressing these challenges systematically. This now includes suggestions for future research directions and practical approaches that could help overcome these barriers. You can find these revisions in the updated manuscript on **pages 16-18**.Also revised in abstract and concusion according. | Page 6-7, [results] part, line 18 begin.  Page 17-18, [The challenges og IoT mobile app for diabetic elderly healthcare services] part, line 1 begin. |
| 3. | **Reviewer #2**: The manuscript "Investigating the Application of IoT Mobile App and Healthcare Services for Diabetic Elderly: A Systematic Review" offers valuable insights into the use of IoT mobile applications for managing diabetes in elderly populations. However, the paper exhibits several weaknesses that need to be addressed to improve its clarity and impact. First, while the authors highlight the potential of IoT technologies, the paper lacks depth in terms of real-world case studies or concrete examples demonstrating the successful implementation of these technologies in diabetic care. The lack of empirical data makes it difficult to evaluate the practical applicability of the findings. Adding specific case studies or implementation results would strengthen the claims and provide a more well-rounded discussion. | 1. Thank you for your valuable feedback. I acknowledge the importance of including real-world case studies and examples to illustrate the practical implementation of IoT technologies in diabetic care. In response, I have added relevant case studies and empirical examples to better demonstrate how these technologies have been successfully applied in real-life settings. These additions can now be found in the revised manuscript on **pages 12-15**. I believe this will help strengthen the claims and provide a more well-rounded discussion as per your suggestion. | Page **12-15**, [The application of IoT mobile app for diabetic elderly healthcare services] part. |
| 4. | **Reviewer #2**: Additionally, the review touches on various challenges such as interoperability, user adoption, and data security, but these issues are discussed superficially. There is little detailed exploration of how these challenges can be addressed, or the role of healthcare professionals and policymakers in overcoming them. To improve the manuscript, the authors should delve deeper into potential solutions or frameworks for tackling these barriers, such as designing user-friendly interfaces for elderly patients or ensuring regulatory compliance for data protection. A more structured approach to discussing these challenges would offer greater practical value and make the findings more actionable for stakeholders in the healthcare industry. | Thank you for your valuable feedback. In response, I have significantly expanded the section on the challenges of IoT mobile apps for diabetic elderly healthcare services. The revised discussion now delves deeper into potential solutions, including frameworks for addressing interoperability, user adoption, and data security issues. I have also included an analysis of the critical roles that healthcare professionals and policymakers play in overcoming these barriers. These revisions can be found in the updated manuscript on **pages 16-17** and **page 18** under the discussion section. Additionally, the abstract and conclusion have been revised accordingly. Also revised in abstract and concusion according. | Page 16-17, [The challenges og IoT mobile app for diabetic elderly healthcare services] part. Page 18 [Discussion] part. |
| 5. | **[Journal requirement]:**  [This research was funded by a grant from Universiti Putra Malaysia Geran Putra grant (GP-IPS). NO: [GP-IPS/2023/9772400].].   Please state what role the funders took in the study.  If the funders had no role, please state: "The funders had no role in study design, data collection and analysis, decision to publish, or preparation of the manuscript."  If this statement is not correct you must amend it as needed.  Please include this amended Role of Funder statement in your cover letter; we will change the online submission form on your behalf. | The funders had no role in study design, data collection and analysis, decision to publish, or preparation of the manuscript, now revised in Acknowledge part. (**Page 20**) | Page 20, [Acnowledge] part. |
| 6. | Please ensure that your manuscript meets PLOS ONE's style requirements. | Follow the PLOS ONE's style requirements. | The whole paper. |
| 7. | As required by our policy on Data Availability, please ensure your manuscript or supplementary information. | Follow the data availability requirements. | / |
