# Supplementary material for: Investigating the application of IoT mobile app and healthcare services for diabetic elderly: A systematic review
Source: PLoS One. 2025 Apr 15;20(4):e0321090. doi: 10.1371/journal.pone.0321090 (PMC11999127; doi:10.1371/journal.pone.0321090)
Supplement: Attachment — Submitted filename: 3-Response to Reviewers-[PONE-D-24-34100R1].doc [file pone.0321090.s005.doc]

|  | **[PONE-D-24-34100R1] –**  **[EMID: 94949ef389940f63]** |
| --- | --- |
|  | **RESPONSE TO REVIEWERS – Minor Revision** |

**Response to Reviewer’s Comments**

I am grateful for your insightful comments. I have been able to incorporate changes to reflect all of the suggestions provided by you before. I have also highlighted the changes in the revised manuscript with track changes for your review.

| **No** | **Comments** | **Response** | **Page** |
| --- | --- | --- | --- |
| 1. | **Reviewer #3**: Introductions need to improve with recent work on Diabetes/IOT/Diabetes in Elders like; <https://doi.org/10.1111/hsc.13522>, <https://doi.org/10.3389/fpubh.2022.871575>, <http://dx.doi.org/10.1109/TR.2023.3336330>, <https://doi.org/10.1109/TIT.2023.3340147> | Thank you for your valuable feedback. I have revised the introduction to incorporate recent work on diabetes, IoT, and diabetes in elderly populations, as per your suggestion. Specifically, I have added the following 4 comprensive citations recommended to strengthen the introduction, referring to the **pages [3,4]** which revised. Thank you again for your insightful comments. | 1. Page 3, 4  [Introduction] part, line 3,15,21, 24. |
| 2. | **Reviewer #3**: The table 2 need to cite, form where or what based this inclusion and exclusion selected. | Thank you for your advice, I added a citation in Table 2 to show the source reference, you can see the revision in **page 5-Table 2**. | Page 5, [Study selection] part, table 2. |
| 3. | **Reviewer #3**: Table 3,4,5, 6; each raw need to cite as per journal style [..] | Thank you for your suggestin, I revised and added each row of per journal citation in Table 3,4,5, 6; you can see the changes in page 7 (Table 3), page 9 (Table 4), page 11 (Table 5) and page 15 (Table 6), where highlighted in red. | Page 7 (Table 3), page 9 (Table 4), page 11 (Table 5) and page 15 (Table 6) with track changes revision. |
| 4. | **Reviewer #3**: Li highlighted that elderly patients often lack the technical knowledge to assess the security risks associated with IoT apps (35). Here Li et al. should be there in place of Li. Please check the manuscript for such error. | Thank you for your careful suggestion, I revised based on your advice, You can find the revision in the updated manuscript on **pages 17-line 10**. | Page 17, line 10. [The challenges og IoT mobile app for diabetic elderly healthcare services] part. |
| 5. | **Reviewer #3**: User adoption and usability also present considerable challenges. Elderly patients often face cognitive and physical limitations that can hinder their ability to effectively use IoT technologies. Future solutions must prioritize designing user-friendly interfaces with simplified device operations, larger text, and voice commands. Moreover, providing adequate training and support for elderly patients, as well as healthcare professionals guiding them, is essential to ensure long-term engagement and motivation. Personalized onboarding experiences, as well as ongoing support, can improve adoption rates and usage. This paragraph needs to cite, with appropriate support reference. | Thank you for your important suggestion, I revised and added related citation to this paragraph to support my iead. You can find the revision with citation in the updated manuscript on **pages 19-line 18, 20, 23**. | Page 19, line 18,20,23. [Discussion] part. |
| 5. | **[Journal requirement]:**  Please review your reference list to ensure that it is complete and correct. If you have cited papers that have been retracted, please include the rationale for doing so in the manuscript text, or remove these references and replace them with relevant current references. Any changes to the reference list should be mentioned in the rebuttal letter that accompanies your revised manuscript. If you need to cite a retracted article, indicate the article’s retracted status in the References list and also include a citation and full reference for the retraction notice. | Thank you for your reminder on reference, I checked the reference list and make sure it’s complete and up-to-date. | Page 22, [References] part. |
